# Large genomic introgression blocks of *Phaseolus parvifolius* Freytag bean into the common bean enhance the crossability between tepary and common beans

**DOI:** 10.1002/pld3.470

**Published:** 2022-12-13

**Authors:** Santos Barrera, Jorge C. Berny Mier y Teran, Juan David Lobaton, Roosevelt Escobar, Paul Gepts, Steve Beebe, Carlos A. Urrea

**Affiliations:** ^1^ Department of Agronomy and Horticulture University of Nebraska Lincoln Nebraska USA; ^2^ Department of Plant Sciences University of California Davis California USA; ^3^ Department of Evolutionary Biology National Australian University Canberra Australia; ^4^ International Center for Tropical Agriculture (CIAT) Palmira Colombia

**Keywords:** embryo rescue, haplotype blocks, interspecific, introgressions, recombinant populations, whole‐genome sequencing

## Abstract

The production of the common bean ( *Phaseolus vulgaris* L.), one of the most important sources of protein and minerals and one of the most consumed grain legumes globally, is highly affected by heat and drought constraints. In contrast, the tepary bean ( *Phaseolus acutifolius* A. Gray), a common bean‐related species, is adapted to hot and dry climates. Hybridization to introduce complex traits from the tepary bean into the common bean has been challenging, as embryo rescue is required. In this study, we report three novel interspecific lines that were obtained by crossing lines from prior common bean × tepary bean hybridization with *Phaseolus parvifolius* Freytag in order to increase the male gametic diversity to facilitate interspecific crosses. These interspecific lines enhanced the crossability of the common bean and tepary bean species while avoiding the embryo rescue process. Crossing these three interspecific lines with tepary beans resulted in 12‐fold more hybrid plants than crossing traditional common beans with tepary beans. Whole‐genome sequencing analysis of these three interspecific lines shows large introgressions of genomic regions corresponding to *P. parvifolius* on chromosomes that presumably contribute to reproductive barriers between both species. The development of these lines opens up the possibility of increasing the introgression of desirable tepary bean traits into the common bean to address constraints driven by climate change.

## 1 INTRODUCTION

The common bean (*Phaseolus vulgaris* L.) is one of the most highly consumed legumes globally, yet climate change threatens its ability to adapt to abiotic and biotic stressors (Beebe, 2012). In the Americas and Africa, the common bean is an essential source of protein, carbohydrates, and minerals (Beebe, 2012; Broughton et al., 2003). The production of common beans relies mainly on small‐farm agriculture, where farmers depend on crop production for their livelihood (Beebe, 2012; Broughton et al., 2003). However, due to abiotic and biotic constraints, common bean yield is relatively low in Latin America and Africa, where it seldom exceeds 900 kg ha^−1^ (Beebe, 2012). Furthermore, common bean production is negatively affected by heat and drought, which have increased due to climate change. These constraints further threaten common bean production in Latin America and Africa (Beebe et al., 2013; Ramirez‐Cabral et al., 2016).

One potential solution to improve the common bean's ability to tolerate warmer and drier conditions is the introgression of tepary bean traits (*Phaseolus acutifolius* A. Gray: Dohle et al., 2019; Moghaddam et al., 2021). Of the five domesticated *Phaseolus* species, the tepary bean is the species with the strongest drought and heat tolerance (Dohle et al., 2019). It is a desert‐adapted species, which is related to the common bean, and has a unique array of abiotic tolerance traits (Pratt & Nabhan, 1988). These traits include heat tolerance (Porch et al., 2021; Traub et al., 2018) and water use efficiency (Markhart, 1985; Polania et al., 2020). However, crossing common bean with tepary bean is challenging due to reproductive isolation barriers between the two species (Gaur et al., 2009; Waines et al., 1988). To obtain viable hybrid plants in common bean/tepary bean crosses requires numerous rounds of pollination, embryo rescue, tissue culture, and backcrosses to alternate parents (congruity backcrossing: Haghighi & Ascher, 1988; Mejía‐Jiménez et al., 1994). Embryo rescue, an in vitro culture technique used to help developing plant embryos that will not survive in vivo, minimizes the hybridization to individual plants rather than large recombinant populations (Waines et al., 1988). In addition, alternating backcrosses limit the possibility of introducing significant variation from the tepary bean to the common bean (Mejía‐Jiménez et al., 1994).

Although the interspecific hybridization of tepary and common beans has been conducted since 1956, few efforts have resulted in advantageous common bean/tepary bean lines. Most of the successful introgression studies have been focused on moving the resistance to common bacterial blight from the tepary bean to the common bean (McElroy, 1985; Singh et al., 2001; Thomas & Waines, 1984). Very few introgression studies have been focused on moving the drought tolerance from tepary beans to common beans (Souter et al., 2017), and no studies have reported a successful introduction of heat tolerance from tepary beans to common beans.

To address this gap, we crossed several tepary beans with elite common bean lines, but our initial effort was hindered by the high level of reproductive incompatibility between both species. However, one cross, which combines three species, *P. vulgaris*, *P. acutifolius*, and *P. parvifolius* resulted in three interspecific lines with enhanced crossability between tepary and common beans while avoiding embryo rescue; furthermore, they produced a large number of hybrids compared to earlier crosses.

In this research, we sought to increase the crossability of *vulgaris*–*acutifolius* crosses by broadening male gametic diversity. Earlier research by Wall and York (1960) and Pratt et al. (1985) had shown that gametic diversity obtained by using pollen of F1 or other hybrid plants instead of pure lines in *Phaseolus* and *Cucurbita* increased crossability in these two genera. To increase gametic diversity in *P. acutifolius*, we chose to hybridize it with *P. parvifolius*, a species closely related to the tepary bean. Indeed, its habitat overlaps partially with that of the tepary bean, and it is morphologically similar to *P. acutifolius* var. *tenuifolius* (Freytag & Debouck, 2002). It belongs to the tepary bean's secondary gene pool (Dohle et al., 2019; Zink & Nagl, 1998). Thus, this species gives partially viable hybrids when it is crossed with *P. acutifolius* accessions (Freytag & Debouck, 2002). AFLPs and microsatellite molecular markers have shown a very close genomic relationship between *P. parvifolius* and *P. acutifolius* (Muñoz et al., 2006; Zink & Nagl, 1998).

In this study, we further confirm the close relationship between *P. acutifolius* and *P. parvifolius* using nuclear genome sequencing data. In addition, we describe the development of three interspecific lines resulting from a triple interspecific cross (*vulgaris*–*acutifolius*–*parvifolius* [VAP]) and the analysis of their whole‐genome sequencing (WGS). The WGS results revealed large introgression blocks of *P. parvifolius* in the genomes of the three interspecific lines, which may explain the increased crossability between tepary and common beans, obviating the need for embryo rescue. Moreover, the results demonstrate why these three interspecific lines can be used potentially as bridge parents for introducing desirable, but genetically complex, traits such as heat and drought tolerance from tepary beans to common beans.

## 2 MATERIALS AND METHODS

### 2.1 Initial interspecific common–tepary bean hybridization

Initially, 11 Mesoamerican common bean lines were crossed with eight tepary bean genotypes and one *P. parvifolius* accession, which had been identified after a field screening under hot and humid conditions in northern Colombia (Supplementary Table S1). Crosses were carried out in the field at the International Center for Tropical Agriculture (CIAT) in Palmira, Colombia, in 2015, following the procedures described by Bliss (1980). Each parental combination included 50 manual pollinations. The common bean lines were used as female parents, whereas tepary beans and the *P. parvifolius* accession were used as male parents. Because early flower abscission and pod abortion occurred, embryos were rescued by removing immature pods from the plant before they abscised. Thirty‐six immature pods from eight crosses were removed approximately 5–7 days after pollination and carried to the tissue culture laboratory at CIAT to conduct embryo rescue. All pods were approximately 3–5 cm long at the time of removal.

### 2.2 Embryo rescue

One hundred fifty‐two embryos were rescued using the method of Mejía‐Jiménez et al. (1994), except that the gelling agent consisted of .2% Gelrite instead of .6% agarose. Briefly, the pods were sterilized for 1 min using 70% ethanol, followed by a 6‐min treatment with 2.5% sodium hypochlorite. Next, the pods were washed three times with sterile deionized water and dissected to extract embryos from the seeds. The embryos were then transferred to a modified MS medium (Murashige & Skoog, 1962) that was prepared as described by Mejía‐Jiménez et al. (1994). Next, the embryos were moved first to a growth chamber (25 ± 1°C and 75% relative humidity) for 2 weeks and then to a simulated photoperiod room with 12‐h dark and 12‐h light (1000 lux) until their primary leaves were fully expanded and their roots had initiated their development.

### 2.3 Plant hardening and fertility recovering

Eighty‐seven fully developed plants from the eight hybrid combinations were transplanted from in vitro tubes into pots with a soil–sand mixture in a 2:1 proportion by weight. The pots were placed in a growth chamber with a continuous nebulization water supply, where the maximum temperature did not exceed 20°C. One week later, the plants were moved to a greenhouse (26°C daytime and 19°C nighttime temperature). In the first round of crosses, only three hybrid plants from the cross between INB834 (female parent) and G40264 (*P. parvifolius*) (male parent) survived. These hybrids were self‐sterile and were subsequently backcrossed with INB 834 or crossed with INB 841 (three‐way cross) (Supplementary Figure S1). The two INB lines (INB834 and INB841) used in this study are interspecific common bean/tepary bean lines that resulted from fertile crosses between the interspecific lines INB605 and INB108, which have been reported as tolerant to drought stress (Beebe et al., 2012). INB605 and INB108 are interspecific lines that resulted from the congruity backcrosses and embryo rescue between *P. vulgaris* and *P. acutifolius* published by Mejía‐Jiménez et al. (1994) and Muñoz et al. (2004). After the second round of crosses, early flower abscission was observed again, and only five pods could be recovered for embryo rescue. Twenty embryos were collected from these pods, but only three hybrid self‐sterile plants were obtained. Since fertility was not fully recovered after the second round of crosses, a third round of alternate crosses with INB lines, used as male parents, was needed to fully recover the fertility (Supplementary Figure S1). After fertility was recovered, 132 seeds were advanced under greenhouse conditions for three generations of inbreeding (F_4_) (Supplementary Figure S1).

### 2.4 Identification of the VAP lines

One hundred thirty‐two families from the F_4_ generation were planted in 2017 in a net‐covered screenhouse in Santander de Quilichao, Colombia (26°C daytime and 19°C nighttime temperature). To introduce a tepary bean background in these interspecific families, 16 of these families were crossed with four male tepary accessions (one cultivar: TARS‐TEP22 [Porch et al., 2013]; three gene bank accessions: G40019, G40068, and G40119). Most of the pollinations either aborted or produced pods without seeds. However, in three families, the pollinations did not abort, and pods and seeds successfully matured on the plant. This result suggested that these families could be crossed directly with tepary beans, thereby avoiding the embryo rescue step. These families were named as experimental lines VAP1, VAP2, and VAP3; VAP stands for *P. vulgaris*, *P. acutifolius*, and *P. parvifolius*.

To corroborate that these three VAP lines could be crossed with tepary beans without embryo rescue and used as bridge lines, we crossed them with 22 different wild and domesticated (landraces and improved lines) tepary beans (used as the males) (Supplementary Table S2). To compare the crossability of the VAP lines with other common bean lines, we crossed the same set of tepary beans with common beans (Supplementary Table S2). Approximately 8–12 pollinations were made per cross‐combination. All pods from crosses with the VAP lines reached maturity. On the other hand, all pods from crosses with common bean genotypes exhibited early abscission and were, therefore, taken to the laboratory for embryo rescue. To confirm hybridization, hybrid plants from crosses with VAP lines as well as common beans were morphologically characterized using the traits listed in Supplementary Table S3.

### 2.5 WGS study and reference genome mapping

To identify the genomic regions that enhance the crossability of the VAP lines with tepary beans, we conducted a WGS analysis of the VAP line parents (INB841, INB834, and G40264) and the three VAP lines (VAP1, VAP2, and VAP3). Due to high phenotypic variability, the WGS analysis was conducted in duplicate on the VAP lines. The WGS reads of G19833 (a bean line belonging to the Andean domesticated gene pool), G40001 (tepary bean), and RWR 719 (a Mesoamerican domesticated bean) were added as outgroup and introgression controls from the study published by Lobaton et al. (2018). The DNA extraction was performed following the methodology reported by Ariani et al. (2016). The WGS analysis was conducted at the genome center of the University of California, Davis, on an Illumina HiSeq 4000, with 100‐bp single‐end fragment length. FastQC (Andrews, 2010) was used to analyze the quality of the DNA sequence reads, and Trimmomatic (Bolger et al., 2014) was used to trim the adaptors and low‐quality reads. The reads were mapped to the latest reference genomes *P. vulgaris* G19833 Andean (version 2.1, Schmutz et al., 2014; Phytozome Version 13: https://phytozome-next.jgi.doe.gov/info/Pvulgaris_v2_1) and *P. acutifolius* G40001 (Moghaddam et al., 2021; wild tepary: Phytozome Version 13: https://phytozome-next.jgi.doe.gov/info/PacutifoliusWLD_v2_0; domesticated tepary: Phytozome Version 13: https://phytozome-next.jgi.doe.gov/info/Pacutifolius_v1_0), using “Bowtie2” (Langmead & Salzberg, 2012).

### 2.6 Interspecific introgression assignation

The identification of sequence variants was performed with the “Next Generation Sequencing Experience Platform” (NGSEP), using the “FindVariants” command with a Q40 quality score (Duitama et al., 2014). The resulting SNP matrix was filtered, leaving only sites where all the samples have SNPs data using the “VCFFilter” module with the –m option. The genotype classification was calculated using the “DistanceMatrixCalculator” command, which uses identical‐by‐state (IBS) algorithms to address relatedness among individuals (Duitama et al., 2014; Tello et al., 2019). Genotypes were visualized with Flap‐Jack (Milne et al., 2010). The interspecific introgression analysis was conducted with the “VCFIntrogression Analysis” module of NGSEP using 100 SNPs windows analysis, following the methodology described by Lobaton et al. (2018). Introgression was assigned if more than 80% of the SNPs were identical to one of the parental lines. In this analysis, we used G40264 as the *P. parvifolius* haplotype reference and G19833 as the *P. vulgaris* haplotype reference. The shared alleles were calculated based on the overall window similarities to G19833 and G40264 haplotypes. By doing this, we were able to determine the location of the interspecific introgressions and how these introgressions differed among the VAP lines and the INB lines.

## 3 RESULTS

### 3.1 Close phylogenetic relationship between *P. acutifolius* and *P. parvifolius* based on genome sequence data

**FIGURE 1:**
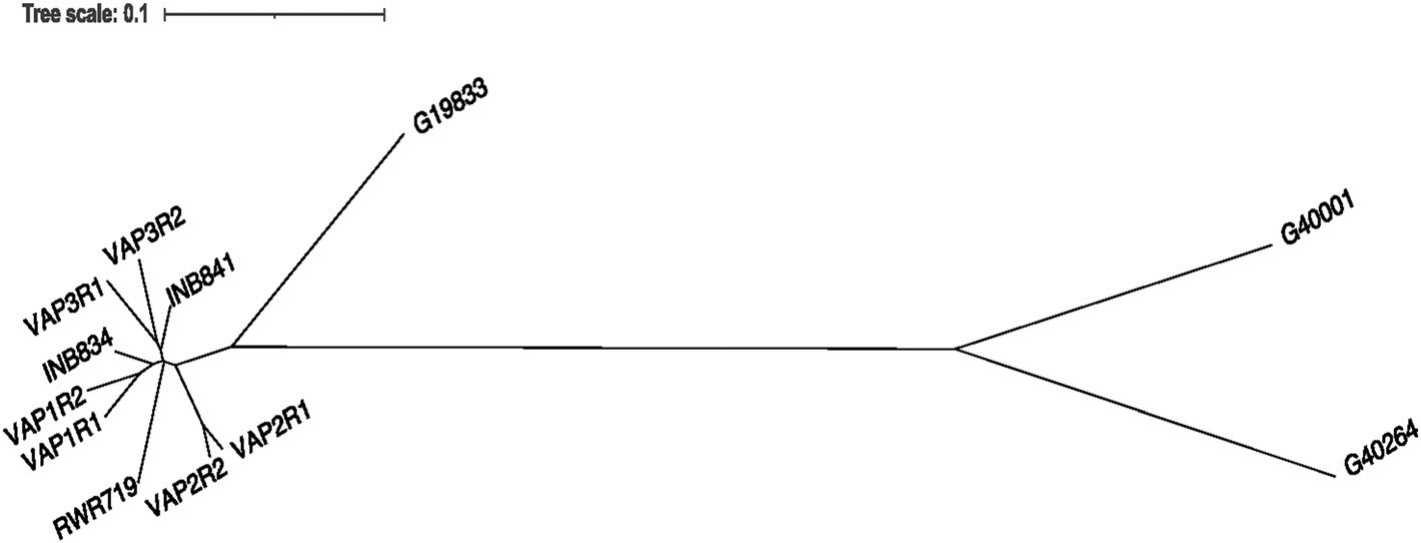
Relatedness tree based on the distance matrix between the interspecific lines (VAP and INB lines), the tepary bean genotype (G40001), the common bean genotype (RWR719), and the *Phaseolus parvifolius* genotype (G40264). The distance matrix was calculated, and the plot was created in the software TASSEL v.5.0 (Bradbury et al., 2007).

The choice of the *P. parvifolius* accession (G40264) to increase gametic diversity was dictated by its phylogenetic closeness to *P. acutifolius* and its agronomic quality, namely, its tolerance to humid heat stress in a field trial on the Caribbean coast of Colombia (S. Barrera, unpublish. results). The percentage of the whole genome sequence reads of *P. parvifolius* mapped to the *P. acutifolius* genome reference was 97% (Table 1), whereas the percentage of whole genome sequence reads of *P. acutifolius* assigned to *P. parvifolius* was 98% (Table 2), confirming a close genomic relationship between these two species Additionally, the genetic distance matrix tree (Figure 1) shows that there was a closer genomic relationship between *P. acutifolius* (G40001) and *P. parvifolius* (G40264) than *P. acutifolius* and the interspecific lines (VAP and INB lines) and a common bean line (RWR719).

**TABLE 1:** Alignment percentage of the whole genome sequence reads to the tepary bean reference genome (G40001) and Andean common bean reference genome (G19833)

Genotype	Genotype group	Total reads	Mapping percentage (%) to the common bean reference genome	Mapping percentage (%) to the tepary bean reference genome
VAP1_R1	Bridge line	41,280,074	96.6	88.8
VAP1_R2	Bridge line	48,043,157	97.2	89.0
VAP2_R1	Bridge line	44,831,917	96.1	89.8
VAP2_R2	Bridge line	34,774,461	96.7	89.7
VAP3_R1	Bridge line	40,358,828	96.7	88.5
VAP3_R2	Bridge line	34,909,203	96.0	89.2
INB841	Interspecific	36,755,518	97.1	88.9
INB834	Interspecific	41,323,904	97.1	88.4
RWR719	Common bean (Mesoamerican)	41,877,662	98.0	86.0
G19833	Common bean (Andean)	331,924,075	97.0	81.4
G40001	Tepary bean (*Phaseolus acutifolius*)	32,999,285	68.2	82.3
G40264	Tepary bean (*Phaseolus parvifolius*)	37,433,199	88.4	96.8

**TABLE 2:** Interspecific introgressions: Number of genomic regions and percentage assigned to either *Phaseolus parvifolius* or *Phaseolus vulgaris* whole genome sequence reads

Genotype	Genotype group	*P. parvifolius G*enome	*P. vulgaris* genome	*P. parvifolius* (%)	*P. vulgaris* (%)	Unmapped	Unmapped (%)	Hetero‐zygous	Hetero‐zygous (%)
G19833	*P. vulgaris* (Andean)	–	30,693	–	100	–	–	–	–
G40001	*Phaseolus acutifolius*	30,132	–	98.2	–	–	–	561	1.8
RWR719	*P. vulgaris* (Mesoamerican)	3	28,942	0.0	94.3	684	2.2	1064	3.5
VAP1_R1	Bridge line	1400	17,806	4.6	58.0	9318	30.4	2169	7.1
VAP1_R2	Bridge line	1658	18,605	5.4	60.6	8091	26.4	2339	7.6
VAP2_R1	Bridge line	1990	17,547	6.5	57.2	10,157	33.1	999	3.3
VAP2_R2	Bridge line	1948	11,704	6.3	38.1	16,291	53.1	750	2.4
VAP3_R1	Bridge line	1618	18,289	5.3	59.6	8381	27.3	2405	7.8
VAP3_R2	Bridge line	2264	12,432	7.4	40.5	12,948	42.2	3049	9.9
INB841	Interspecific	97	17,041	0.3	55.5	12,579	41.0	976	3.2
INB834	Interspecific	280	19,859	.9	64.7	9530	31.0	1024	3.3
G40264	*P. parvifolius*	30,693	–	100.0	–	–	–	–	0.0

### 3.2 Initial interspecific hybridization, embryo rescue, and plant hardening

To introduce genetic variation from the tepary bean to the common bean, we crossed 11 common bean elite experimental lines with eight *P. acutifolius* accessions and one *P. parvifolius* accession (Supplementary Table S1). Hybridization between *P. vulgaris*, *P. acutifolius*, and *P. parvifolius* was challenging as strong reproductive incompatibility was present. In eight out of 11 initial crosses, 93% of pollinated flowers abscised early or produced empty pods, and 136 embryos were rescued from 36 immature pods. From the 136 rescued embryos, only three plants from the cross with G40264 (*P. parvifolius*) survived the hardening step. These three plants represent a hybridization rate of .005 plants per pollination from the 550 pollinations that were made in the initial 11 crosses. The three plants were self‐sterile, and it was necessary to make two consecutive crosses using INB lines as the male parent to fully recover fertility, which resulted in 132 inbred interspecific F_4_ families (Supplementary Figure S1).

The initial challenges experienced while crossing tepary and common beans confirm the reproductive barriers observed previously between the two species (Andrade‐Aguilar & Jackson, 1988; Mejía‐Jiménez et al., 1994; Mok et al., 1978; Parker & Michaels, 1986; Smartt, 1970). Moreover, the observation that most pollinated flowers, from the cross between common bean and *P. parvifolius*, abscised early suggests a high level of reproductive incompatibility between these two species. Reproductive incompatibility between common bean and *P. parvifolius* had previously been overcome by embryo rescue and congruity backcrosses (Singh et al., 1998). However, in a study reported by Rao et al. (2013), these interspecific lines obtained with congruity backcrosses did have a comparative advantage in their drought tolerance response because of the dilution of the *P. parvifolius* contribution caused by the congruity backcrossing and the use of the drought‐susceptible common bean parent, ICA Pijao.

### 3.3 Identification of the VAP lines

We observed early flower abscission and empty pods when we crossed 16 interspecific F_4_ families with four male tepary beans. However, in three families (which we named VAP1, VAP2, and VAP3), three pollinated flowers per family did not abscise early, and the pods reached maturity in the plant, leading to 12 mature seeds in total. The hybrid seeds were viable when planted and produced hybrid self‐sterile plants, which confirmed the hybridization. This phenomenon was unusual because we had not obtained mature seeds in any of the previous direct crosses with tepary beans. Only five studies have reported the obtention of hybrid mature F_1_ seeds between common and tepary beans (Ferwerda et al., 2003; Haghighi et al., 1984; Park et al., 1986; Smartt, 1970; Thomas et al., 1983). The ability to produce hybrid seeds from crosses between tepary beans and lines with a high level of common bean ancestry represents a vital breakthrough to overcoming reproductive barriers between the two species.

**FIGURE 2:**
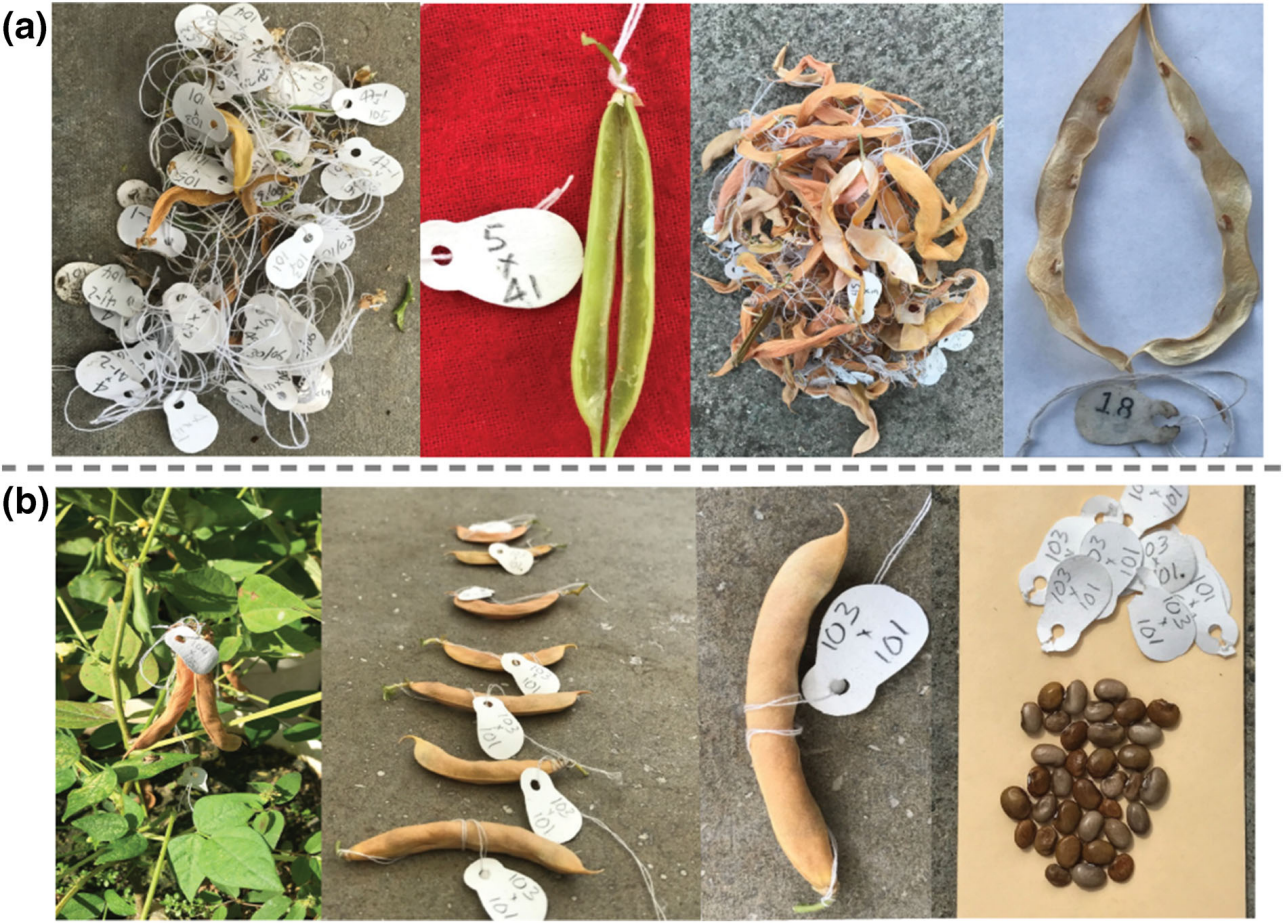
Interspecific hybridization between common bean and tepary bean. (a) Flowers abscised at a very early stage and pods without seeds, from crosses between tepary beans and common beans. (b) Mature pods and seeds from crosses between the VAP lines and tepary beans

**FIGURE 3:**
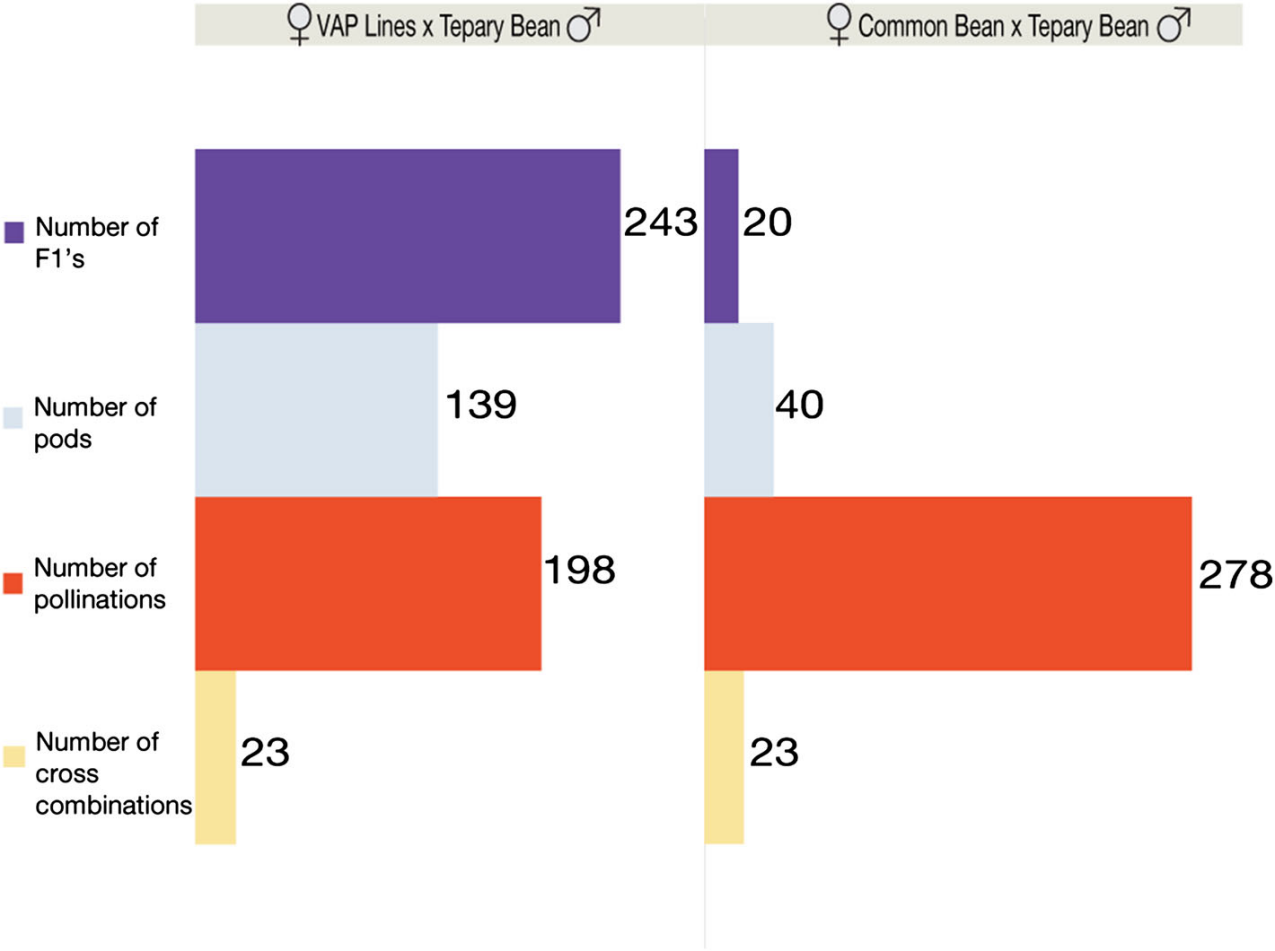
Comparison of the ability to cross tepary beans with traditional common bean lines and the interspecific VAP lines. Left) 22 crosses of VAP lines (used as female parents) with tepary beans (used as male parents). Right) 23 crosses of traditional common beans (female parents) with tepary beans (male parents)

To confirm that VAP lines allowed fertile crossing between common and tepary beans without the need for embryo rescue, we created two sets of crosses under greenhouse conditions. One set of crosses used VAP lines (as female parents) and tepary beans (as male parents), and the other set used common beans (as female parents) and tepary beans (as male parents). Unsurprisingly, common beans that were fertilized with pollen from tepary beans showed premature shedding of flowers, and pods did not develop seeds (Figure 2a). From 278 pollinations between common and tepary beans, no pods reached maturity, and 40 flowers produced immature pods. Moreover, only 20 hybrid plants were obtained with embryo rescue (Figure 3). Conversely, when we crossed the VAP lines with tepary beans, a substantial hybridization success was observed. From 198 pollinations, 139 pods reached maturity in the plant, and 243 hybrid seeds were harvested from these pods (Figure 2b, Figure 3). From the hybrid seeds obtained, 39% corresponded to VAP1, 35% to VAP2, and 26% to VAP3. Overall, a substantial difference in the hybridization rate was observed for crosses between common bean and VAP lines with tepary beans. Specifically, common beans had a hybridization rate of .071 plants per pollination, whereas the hybridization rate for VAP lines was 1.23 plants per pollination.

All the interspecific hybrids obtained from crosses with VAP lines and common bean lines were self‐sterile and showed intermediate common–tepary bean morphological characteristics (Supplementary Table S3). The fertility was fully recovered when the hybrids were crossed once with pollen of common bean lines (Supplementary Figure S2). Although embryo rescue was not required for the hybridization between the VAP lines and tepary beans, self‐sterility in the hybrids indicates that there is still a limitation in using these lines, and there is a need for an additional cross with a fertile common bean parent to recover full fertility.

### 3.4 WGS and sequence read mapping

To elucidate the genomic constitution of the VAP lines and identify genomic regions that could be responsible for enhancing VAP lines' ability to cross with tepary beans, we conducted WGS. We aligned the whole genome sequences of VAP lines with the tepary bean (*P. acutifolius* = G40001) and common bean (*P. vulgaris* = G19833) reference genomes and determined differences in the mapping percentage. The VAP lines, interspecific parents, and common bean controls generally had a higher alignment rate with the common bean reference (97%) than the tepary bean reference (88%) (Table 1). This result suggests that, overall, the VAP lines and interspecific parents are more related to the common bean than the tepary bean. Given that the VAP lines were backcrossed to INB lines that generally have over 90% common bean background (Lobaton et al., 2018), we expected a higher percentage alignment between VAP lines and the common bean reference compared to the tepary bean reference.

The *P. parvifolius* parental accession (G40264) had an alignment rate of 88% with the common bean reference genome and 97% with the tepary reference genome (Table 1). Due to the close phylogenetic relationship between G40264 and the tepary bean reference genome (G40001) (Freytag & Debouck, 2002), a higher percentage alignment between G40264 with tepary bean compared to common bean was also expected. The VAP lines had, on average, a 3% higher overall alignment with the tepary bean reference (G40001) than the common bean control (RWR719), and an 8% higher overall alignment than the Andean common bean reference (G19833) (Table 1).

### 3.5 Interspecific introgressions mapping

To identify the genomic introgressions that could be critical in enhancing the crossability between the VAP lines and tepary beans, we mapped the genomic regions that corresponded to *P. parvifolius* and *P. vulgaris*. We found that the VAP lines had more genomic regions corresponding to the *P. parvifolius* genome than the INB parental lines and the common bean controls. While less than 1% of the *P. parvifolius* genomic regions were found in the INB lines, 4.6%–7.4% of these genomic regions were found in the VAP lines (Table 2), which indicates that the VAP lines have more interspecific introgressions in their genomes than the INB lines. Also, the VAP lines had on average 930 more heterozygous genomic regions than the INB lines and the common bean controls, indicating that those introgressions need to be fixed or recombined (Table 2). We also found that *P. parvifolius and P. acutifolius* share 98% of the 30,693 mapped genomic regions (Table 2), confirming the close genetic relationship between both species (Freytag & Debouck, 2002).

**FIGURE 4:**
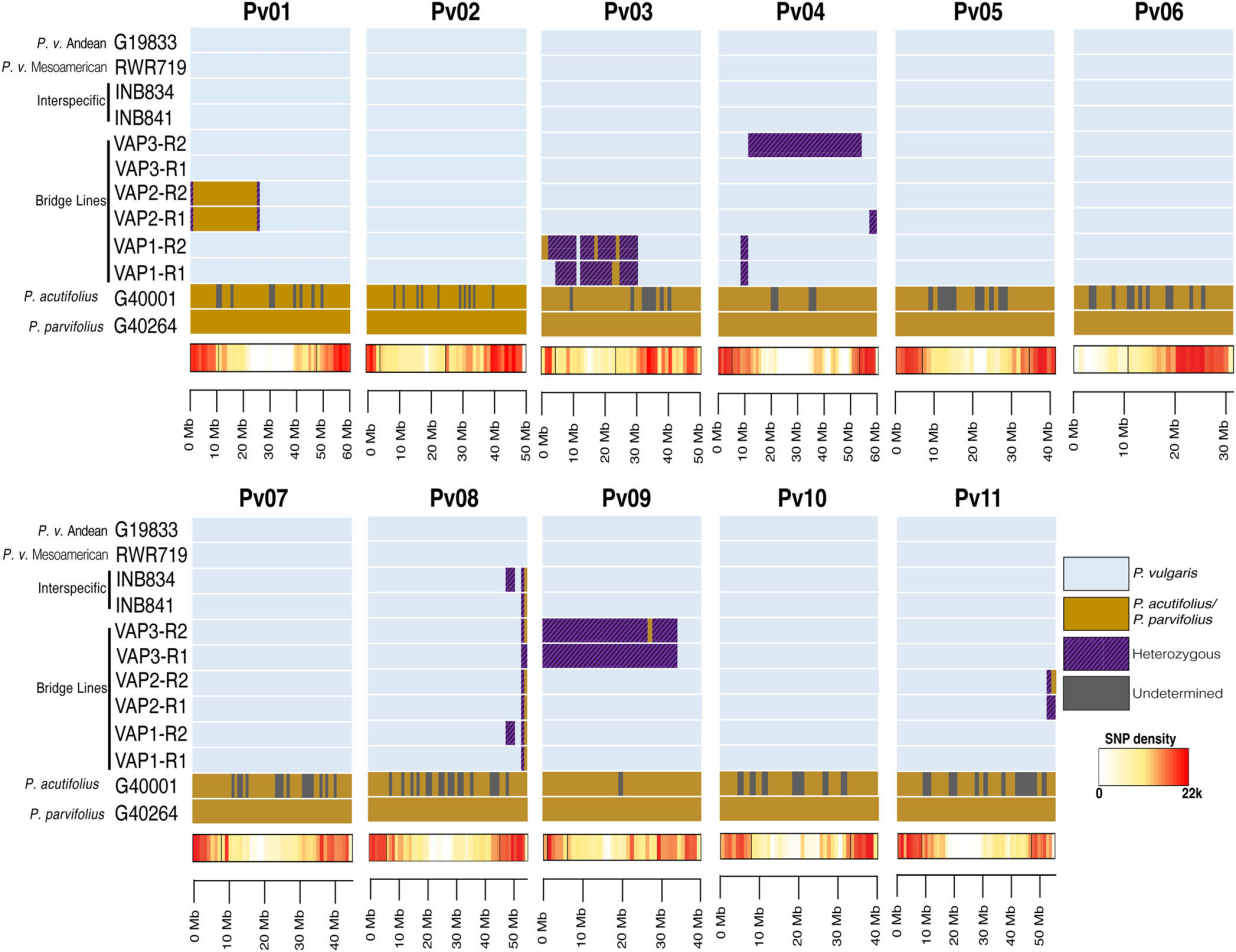
Mapping of the interspecific introgressions in the VAP lines, INB lines, two common bean genotypes (Andean and Mesoamerican), and two tepary bean genotypes in the 11 chromosomes. Light blue indicates the common bean background. Brown indicates the tepary bean background. Purple indicates heterozygous SNPs. The heat map shows the SNP density. G40001, which is the tepary bean reference genome is presented in this figure only to compare *Phaseolus parvifolius* and *Phaseolus acutifolius* genomes.

The introgression analysis also revealed large *P. parvifolius* haplotype blocks in the VAP lines that are not present in the INB lines and the common bean controls. Specifically, VAP1 has large heterozygous introgression blocks spanning over 28 Mb, and small homozygous haplotype blocks spanning over 1.2 Mb, both on chromosome Pv03. It also has smaller heterozygous as well as homozygous introgression blocks on chromosomes Pv04 and Pv08 (Figure 4). VAP2 has a large homozygous *P. parvifolius* haplotype block of more than 39 Mb on chromosome Pv01 and a small (35 K) homozygous introgression block on chromosome Pv08 (Figure 4). It also has heterozygous introgression blocks on chromosomes Pv04, Pv08, and Pv011 (Figure 4).

**FIGURE 5:**
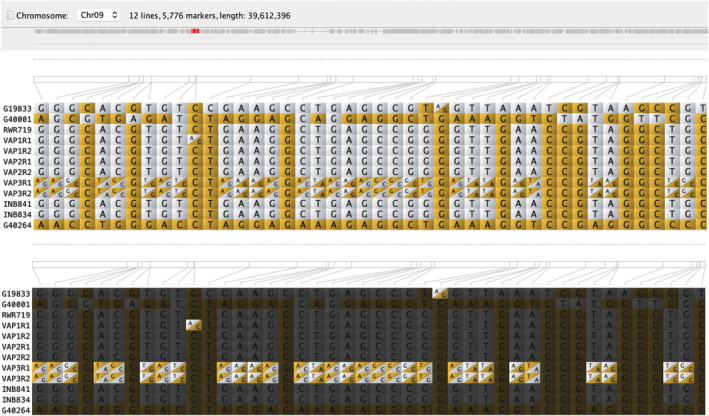
Flapjack visualization of introgressions from *Phaseolus parvifolius* (G40264) in the VAP lines in chromosome Pv09. Highlighted heterozygous introgression regions in the line VAP3 in replication 01 (R1) and replication 02 (R2). Heterozygous regions are defined as regions that share nucleotides from both reference haplotypes (G19833 and G40264). G40001, which is the tepary bean reference genome is presented in this figure only to compare *P. parvifolius* and *Phaseolus acutifolius* genomes.

VAP3 is the bridge line that has the most heterozygous introgression blocks of the three VAP lines (Figure 5). Interestingly, nearly the entire chromosome Pv09 (~36 Mb) is composed of heterozygous SNPs from *P. parvifolius* and *P. vulgaris* (Figures 4 and 5). VAP3 also has a large heterozygous block (54 Mb) in replication 2 (R2) on chromosome Pv04, which is not present in replication 1 (R1) (Figure 3), indicating that this line is not entirely fixed. Interestingly, the introgressions we found on chromosome Pv08 were also found by Lobaton et al. (2018) in the VAX and INB lines, indicating that these tepary introgressions are conserved across multiple interspecific lines. However, unlike the VAP lines, the INB lines do not have more interspecific tepary haplotype blocks on the other chromosomes. Also, as expected, the unintrogressed control common bean line RWR719 did not show any introgressions (Figure 4). Still, three genomic regions were assigned to the *P. parvifolius* group (Table 2), which indicates that small genomic regions assigned to *P. parvifolius* cannot necessarily be considered interspecific introgressions but reflect a common ancestry in the genus *Phaseolus*.

## 4 DISCUSSION

In this study, we have confirmed that crossing the traditional common bean (i.e., without tepary introgressions) with tepary bean is challenging. Although hybridization is possible with embryo rescue, it limits efficient interspecific introgression. Similar challenges have been reported in previous common and tepary bean hybridization studies (Andrade‐Aguilar & Jackson, 1988; Mok et al., 1978; Mejía‐Jiménez et al., 1994; Parker & Michaels, 1986; Pratt et al., 1985; Rabakoarihanta et al., 1979; Souter et al., 2017; Thomas et al., 1983). Although different hybridization strategies such as alternating female genotypes (Parker & Michaels, 1986; Rabakoarihanta et al., 1979) or congruent backcrossing have been attempted (Anderson et al., 1996; Haghighi & Ascher, 1988), only a few hybrid plants are usually obtained. Here, we demonstrate that it is possible to improve the crossability of common bean and tepary bean by using three novel interspecific genotypes (VAP lines). These interspecific genotypes allowed us to obtain a large number of interspecific common–tepary bean hybrids without embryo rescue (Figure 3). Strikingly, we were able to achieve up to 12‐fold more hybrids when we crossed these VAP lines with tepary beans than when we crossed traditional common beans with tepary beans and used embryo rescue (Figure 3). We needed 21% fewer pollinations when we crossed VAP lines with the tepary beans than when we crossed common beans with tepary beans (Figure 3).

Five other studies have also reported successful hybridization between common bean and tepary bean without embryo rescue but did not report finding a large number of hybrid seeds (Ferwerda et al., 2003; Haghighi et al., 1984; Park et al., 1986; Smartt, 1970; Thomas et al., 1983). Producing large recombinant populations is fundamental to introducing genetically complex traits, such as heat and drought tolerance, from the tepary to the common bean. Additionally, this is the first study that has achieved interspecific crosses using a large number of different forms of tepary beans, such as wild accessions, landraces, and improved varieties. Our success in increasing crossability may also be due to an increase in gametic diversity, as *P. parvifolius*, a close relative of *P. acutifolius* (Freytag & Debouck, 2002; Figure 1), was included in the crossing scheme. Gametic diversity has been credited with (or has been suggested to achieve) an increase in interspecific crossability in *Phaseolus* and *Cucurbita* (Gepts, 1981; Paris, 2016; Pratt et al., 1985; Wall & York, 1960), rye–wheat (Meister & Tjumjakoff, 1928), and eggplant (Schaff et al., 1982).

Further genomic analysis of the VAP lines, conducted to identify genomic regions that are key in enhancing their crossability, revealed large introgression blocks from *P. parvifolius* to the VAP lines. The inheritance of these large introgression blocks seen in the VAP lines suggests a lack of recombination events in those genomic regions. Some of the introgression blocks found here have been reported in genomic regions where considerable rearrangements between common and tepary beans have occurred (Moghaddam et al., 2021). For instance, a major QTL has been reported, which contributes to the reproductive incompatibility between the two species on chromosome Pv09 (Soltani et al., 2020), which is one of the chromosomes with large common–tepary bean rearrangements (Gujaria‐Verma et al., 2016; Moghaddam et al., 2021). In this study, we saw that chromosome Pv09 was one of the chromosomes with the largest *P. parvifolius* introgression events, suggesting that cross‐compatibility between tepary beans and the VAP3 lines may have been enhanced by this interspecific introgression. In contrast, different introgressions present on chromosomes Pv01, Pv03, Pv04, Pv08, and Pv11, found in the VAP1 and VAP2 lines (Figure 3), could have enhanced their cross‐compatibility with tepary beans since they lacked the Pv09 introgression event.

VAP 3 had a reduced ability to cross with tepary beans (only 26% of the crosses were obtained with VAP3), which could be explained by the high level of heterozygous introgressions of this line. A high number of heterozygous introgression regions, such as the introgressions on chromosome Pv09 (Figures 4 and 5), suggest that it might be challenging to maintain the ability to cross the VAP lines with tepary beans since the lines could segregate in favor of common bean alleles. Therefore, there is a need to continue breeding the VAP lines and fix the heterozygous introgressions.

The only shared introgressed segment between the two INB and three VAP lines, but absent in the two common bean lines, was the short terminal segment observed on chromosome Pv08. This introgression had already been observed by Lobaton et al. (2018) in three VAX lines (VAX 3, 4, and 6), resulting from interspecific hybridization with tepary beans and pyramiding multiple sources of resistance to common bacterial blight, including tepary beans (Singh et al., 2001; Singh & Muñoz, 1999). Given the frequent position of disease resistance genes or clusters in distal chromosome positions (e.g., Kelly et al., 2003; Schmutz et al., 2014), this segment could be a carrier of part of the bacterial resistance exhibited by the VAX lines mentioned. Although the two INB lines included in this study (INB834 and INB841) carried the terminal Pv08 introgression, this is not always the case as Lobaton et al. (2018) showed that INB827 did not carry this introgression. Thus, any putative crossability‐enhancing property attributable to the terminal Pv08 introgression cannot be assigned with certainty to all INB lines. Nevertheless, it is possible that this segment could carry both common bacterial blight resistance and enhanced crossability, either by pleiotropy or tight linkage.

Heterozygosity is still high in the VAP lines, based on the sequence data obtained in this study. Although the pursuit of fixation is highly desirable for the maintenance of the VAP lines, it remains to be seen whether the increased crossability with tepary beans can be maintained in a homozygous state or if the heterozygous state has to be maintained, perhaps by a marker‐assisted selection of heterozygous introgressed chromosome segments.

The development of the novel VAP lines, which allows hybridizing common beans with tepary beans and obtaining large common bean/tepary bean populations, may open up the possibility of efficiently exploiting the tertiary gene pool to move heat and drought tolerance from tepary beans to common beans without the need for embryo rescue and the associated cost and infrastructure.

## CONFLICTS OF INTEREST

The authors declare that they have no conflict of interest related to this manuscript.

## AUTHOR CONTRIBUTIONS

Santos Barrera Lemus developed the VAP lines, aligned the reads to the reference genomes, and wrote the manuscript draft. Jorge C. Berny Mier y Teran conducted the DNA extraction, coordinated the WGS analysis, and edited the manuscript. Juan David Lobaton performed de bioinformatic analysis of the WGS data and introgression analysis. Roosevelt Escobar performed the embryo rescue. Steve Beebe, Paul Gepts, and Carlos A. Urrea supervised the study and edited the final version of the manuscript.

## Supporting information


**Figure S1.** Crossing scheme of the VAP lines. Left) VAP1 crossing scheme. Right) VAP2 and VAP3 crossing scheme. WER = with embryo rescue. WOER = without embryo rescue. VAP2 and VAP3 are sister lines.

**Figure S2** Greenhouse‐grown plants: Left) A fertile (pods indicated with a red arrow) VAP line. Center) An interspecific self‐sterile hybrid (VAP line X G 40019) obtained without embryo rescue. Right) A fertile interspecific hybrid crossed with pollen of a fertile common line [(VAP1 X G 40019)F1 X SEF10], showing that the fertility was recovered (pods indicated with a red arrow).

**Table S1.** Common bean lines and tepary bean genotypes used in the initial interspecific hybridization
**Table S2.** Common bean lines and tepary bean genotypes used to compare crossability between VAP lines and traditional common beans
**Table S3.** Morphological characterization of the tepary‐common bean hybrids

## Data Availability

The Whole Genome Sequence data for this study are available online at the National Center for Biotechnology Information (NCBI) repository https://submit.ncbi.nlm.nih.gov/subs/sra/SUB10611384/. (BioProject ID:PRJNA778000)
