# Supplementary figures and images for: Large genomic introgression blocks of Phaseolus parvifolius Freytag bean into the common bean enhance the crossability between tepary and common beans
Source: Plant Direct. 2022 Dec 13;6(12):e470. doi: 10.1002/pld3.470 (PMC9745863; doi:10.1002/pld3.470)

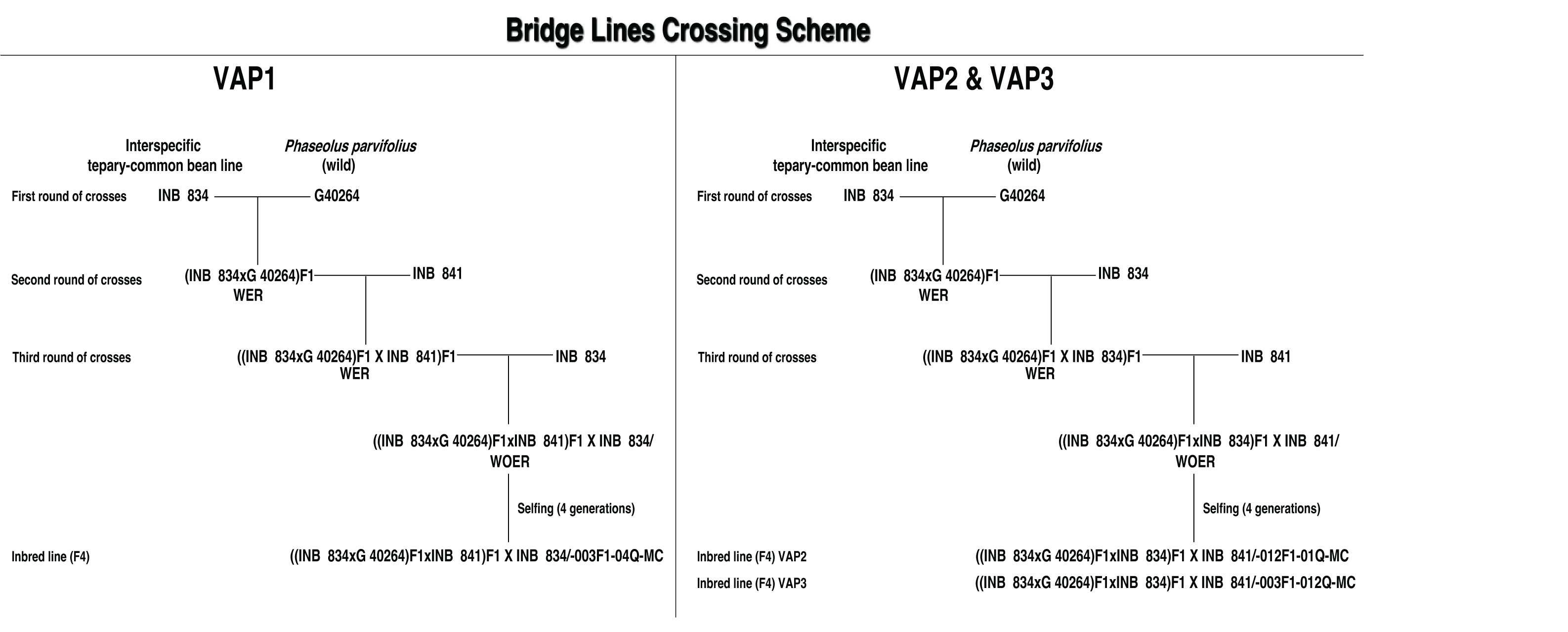

Supplement: Supplementary file 1 — Figure S1. Crossing scheme of the VAP lines. Left) VAP1 crossing scheme. Right) VAP2 and VAP3 crossing scheme. WER = with embryo rescue. WOER = without embryo rescue. VAP2 and VAP3 are sister lines. [file PLD3-6-e470-s003.tiff]

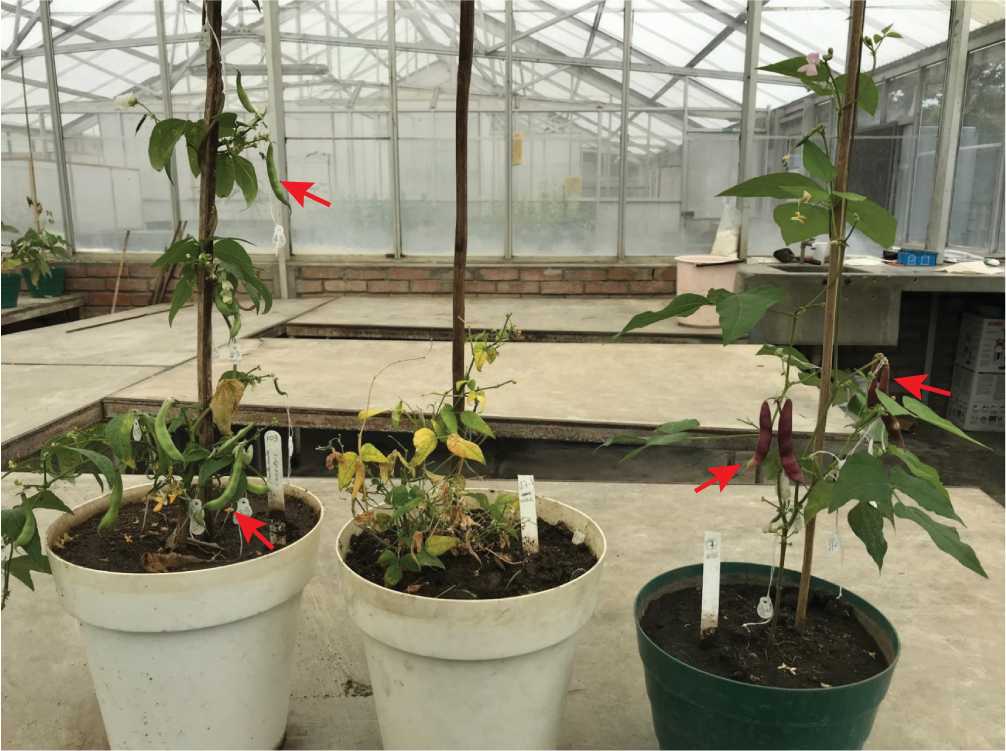

Supplement: Supplementary file 2 — Figure S2 Greenhouse‐grown plants: Left) A fertile (pods indicated with a red arrow) VAP line. Center) An interspecific self‐sterile hybrid (VAP line X G 40019) obtained without embryo rescue. Right) A fertile interspecific hybrid crossed with pollen of a fertile common line [(VAP1 X G 40019)F1 X SEF10], showing that the fertility was recovered (pods indicated with a red arrow). [file PLD3-6-e470-s001.tiff]
