# Supplementary material for: Large genomic introgression blocks of Phaseolus parvifolius Freytag bean into the common bean enhance the crossability between tepary and common beans
Source: Plant Direct. 2022 Dec 13;6(12):e470. doi: 10.1002/pld3.470 (PMC9745863; doi:10.1002/pld3.470)
Supplement: Supplementary file 3 — Table S1. Common bean lines and tepary bean genotypes used in the initial interspecific hybridization Table S2. Common bean lines and tepary bean genotypes used to compare crossability between VAP lines and traditional common beans Table S3. Morphological characterization of the tepary‐common bean hybrids [file PLD3-6-e470-s002.docx]

| **Table S1.** Common bean lines and tepary bean genotypes used in the initial interspecific hybridization | | | | | | | | | | | | | | | | | | |
| --- | --- | --- | --- | --- | --- | --- | --- | --- | --- | --- | --- | --- | --- | --- | --- | --- | --- | --- |
| **Cross #** | **Common beans (female parent)** | **GH** | **PSC** | **SSC** | **SCP** | **SDBR** | **SSZ** |  | **Tepary beans (male parent)** | **Species** | **Form** | **GH** | **PSC** | **SSC** | **SCP** | **SDBR** | **SSZ** | **100SW** |
| 120 | SEN 97 | 2B | 8 |  | 0 | 1 | 2 | x | G 40022 | *P. acutifolius* | Landace | 3B | 3 |  |  | 1 | 1 | 15 |
| 121 | SMR 139 | 2B | 6 |  | 0 | 1 | 2 | x | G 40022 | *P. acutifolius* | Landace | 3B | 3 |  |  | 1 | 1 | 15 |
| 122 | SEF 60 | 2A | 6 |  | 0 |  | 2 | x | G 40027 | *P. acutifolius* | Landace | 3B | 1 |  |  | 1 | 1 | 12 |
| 123 | SMN 57 | 2A | 8 |  | 0 |  | 2 | x | G 40143 | *P. acutifolius* | Landace | 3B | 1 |  |  | 1 | 1 | 13 |
| 124 | SEF 60 | 2A | 6 |  | 0 |  | 2 | x | G 40028 | *P. acutifolius* | Landace | 3B | 1 |  |  | 1 | 1 | 15 |
| 125 | INB 834 | 2A | 4 |  | 0 |  | 1 | x | G 40264 | *P. parvifolius* | Wild | 3B | 2 | 9 |  | 2 | 1 | 1 |
| 126 | INB 834 | 2A | 4 |  | 0 |  | 1 | x | G 40045 | *P. acutifolius* | Wild | 3B | 2 | 9 |  | 2 | 1 | 6 |
| 127 | SMR 132 | 2B | 6 |  | 0 | 3 | 2 | x | G 40261 | *P. acutifolius* var. tenuifolus | Wild | 3B | 2 | 9 |  | 2 | 1 | 2 |
| 128 | SEN 56 | 2A | 8 |  | 0 | 2 | 2 | x | G 40001 | *P. acutifolius* | Landace | 3B | 1 |  |  | 1 | 1 | 16 |
| 129 | SMR 139 | 2B | 6 |  | 0 | 1 | 2 | x | G 40028 | *P. acutifolius* | Landace | 3B | 1 |  |  | 1 | 1 | 15 |
| 130 | SEF 60 | 2A | 6 |  | 0 |  | 2 | x | G 40001 | *P. acutifolius* | Landace | 3B | 1 |  |  | 1 | 1 | 16 |

GH: Growth habit; FLCOL: Flower color; PSC; Primary seed color; SSC: Secondary seed color; SCP: Seed color pattern; SDBR: Seed brilliance; SSZ: Seed size; 100SW: 100 seeds weight

| **Table S2.** Common bean lines and tepary bean genotypes used to compare crossability between VAP lines and traditional common beans | | | | | | | | | | | | | | | | | |  |  |
| --- | --- | --- | --- | --- | --- | --- | --- | --- | --- | --- | --- | --- | --- | --- | --- | --- | --- | --- | --- |
| **Tepary beans** | | | | | | | | | | **Common beans** | | | | | | | | | |
| **ID** | **FLCOL** | **GH** | **PSC** | **SSC** | **SCP** | **SDBR** | **SSZ** | **Specie** | **Form** | **ID** | **FLCOL** | **GH** | **PSC** | **SSC** | **SCP** | **SDBR** | **SSZ** | **100SW** | **BCMNV** |
| G 40019 | 5 | 2B | 8 |  | 0 | 3 | 1 | *P. acutifolius* | Landrace | SMC 214 | 1 | 2B | 7 | 2 | J | 1 | 2 | 30 | 12N |
| G 40036 | 5 | 2B | 8 |  | 0 | 3 | 1 | *P. acutifolius* | Landrace | SMR 155 | 4 | 2A | 6 |  | 0 | 3 | 2 | 32 | 12N |
| G 40068 | 1 | 3B | 3 |  | 0 | 1 | 2 | *P. acutifolius* | Landrace | ICTA LIGERO | 5 | 2A | 8 |  | 0 |  | 1 |  |  |
| G 40084 | 4 | 3B | 2 | 8 | J | 1 | 1 | *P. acutifolius* | Landrace | SEF 10 | 1 | 2A | 6 |  | 0 | 3 | 2 | 26 | 12_O |
| G 40111 | 5 | 3B | 8 |  | 0 | 1 | 1 | *P. acutifolius* | Landrace | SEN 118 | 5 | 2A | 8 |  | 0 | 1 | 2 | 23 | 12N |
| G 40119 | 5 | 3B | 8 |  | 0 | 2 | 1 | *P. acutifolius* | Landrace | INB 834 | 1 | 2A | 4 |  | 0 | 2 | 1 | 26 | 12N |
| G 40168 | 5 | 3B | 9 | 1 | J | 1 | 1 | *P. acutifolius* | Wild | INB 841 | 1 | 2A | 4 | 2 | J | 1 | 1 |  | 11N |
| G 40173A | 1 | 3B | 3 |  |  | 1 | 1 | *P. acutifolius* | Landrace | SMR 139 | 1 | 2B | 6 |  |  | 0 | 3 | 28 | 12_O |
| G 40200 | 4 | 3B | 8 | 1 | J | 3 | 1 | *P. acutifolius* | Landrace | SEN 97 | 5 | 2B | 8 |  | 0 | 1 | 2 |  | 12N |
| G 40284 | 1 | 3B | 1 |  |  | 1 | 1 | *P. acutifolius* | Landrace | SMN 57 | 5 | 2A | 8 |  | 0 |  | 2 |  | 12N |
| TEP 22 | 1 | 2B | 1 |  | 0 | 2 | 1 | *P. acutifolius* | Improved | SMR 132 | 2 | 2B | 6 |  | 0 | 3 | 2 |  | 12N |
| G 40022 | 1 | 3B | 1 |  | 0 | 1 | 1 | *P. acutifolius* | Landrace | SEN 56 | 5 | 2A | 8 |  | 0 | 2 | 2 |  | N |
| G 40056 | 5 |  | 2 | 8 | J | 2 | 1 | *P. acutifolius* | wild | SEF 60 | 1 | 2A | 6 |  | 0 |  | 2 |  | 12N |
| G 40142 |  |  |  |  |  |  |  | *P. acutifolius* | Landrace |  |  |  |  |  |  |  |  |  |  |
| G 40148 |  |  |  |  |  |  |  | *P. acutifolius* | Landrace |  |  |  |  |  |  |  |  |  |  |
| G 40161 | 5 |  | 2 |  | 0 | 2 | 1 | *P. acutifolius* | Landrace |  |  |  |  |  |  |  |  |  |  |
| G 40237A | 1 |  | 5 |  | 0 | 1 | 1 | *P. acutifolius* | Landrace |  |  |  |  |  |  |  |  |  |  |
| G 40264 | 5 | 3B | 2 | 9 | M | 2 | 1 | *P. parvifolius* | Wild |  |  |  |  |  |  |  |  |  |  |
| G 40274 |  |  |  |  |  |  |  | *P. acutifolius* | Landrace |  |  |  |  |  |  |  |  |  |  |
| G 40279 | 5 |  | 1 |  | 0 | 1 | 1 | *P. acutifolius* | Landrace |  |  |  |  |  |  |  |  |  |  |
| G 40287 | 5 |  | 2 | 8 | J | 1 | 1 | *P. acutifolius* | Wild |  |  |  |  |  |  |  |  |  |  |
| TEP 23 | 5 | 2B | 8 |  | 0 | 1 | 2 | *P. acutifolius* | Improved |  |  |  |  |  |  |  |  |  |  |
| GH: Growth habit; FLCOL: Flower color; PSC; Primary seed color; SSC: Secondary seed color; SCP: Seed color pattern; SDBR: Seed brilliance; SSZ: Seed size; 100SW: 100 seeds weight; BCMNV: Bean Common Mosaic Necrosis Virus (N=Necrotic reaction, has I gene; O=No symptoms, has recessive resistance) | | | | | | | | | | | | | | | | | | | |

| **Table S3.** Morphological characterization of the tepary-common bean hybrids | | | |
| --- | --- | --- | --- |
| **Trait** | **VAP lines** | **Tepary bean lines** | **Hybrids VAP lines x *P. acutifolius*** |
| Flowers color | White | White and purple | 25 hybrids white 218 hybrids lavender |
| Bracteole type | Cordate large | Triangular small | Lanceolate small (all) |
| Leaves shape | Ovate | Lanceolate | Hastate (all) |
| Growth habit | Indeterminate bush habit | Indeterminate climbing habit with weak mainstem and prostrate stem and branches | Indeterminate bush habit (all) |
| Fertility | Fertile | Fertile | Sterile (all) |
